# In Situ Dynamics of F-Specific RNA Bacteriophages in a Small River: New Way to Assess Viral Propagation in Water Quality Studies

**DOI:** 10.1007/s12560-016-9266-0

**Published:** 2016-10-22

**Authors:** Blandine Fauvel, Christophe Gantzer, Henry-Michel Cauchie, Leslie Ogorzaly

**Affiliations:** 1 Department of Environmental Research and Innovation (ERIN), Luxembourg Institute of Science and Technology (LIST), 41, rue du Brill, 4422 Belvaux, Luxembourg; 2 Laboratoire de Chimie, Physique et Microbiologie pour l’Environnement (LCPME), UMR 7564, Faculté de Pharmacie, Université de Lorraine, 5 rue Albert Lebrun, Nancy, 54000 France; 3 CNRS, LCPME, UMR 7564, Nancy, 54000 France

**Keywords:** F-specific RNA bacteriophages, In situ propagation modelling, In situ survival, Water, Sediment

## Abstract

The occurrence and propagation of enteric viruses in rivers constitute a major public health issue. However, little information is available on the in situ transport and spread of viruses in surface water. In this study, an original in situ experimental approach using the residence time of the river water mass was developed to accurately follow the propagation of F-specific RNA bacteriophages (FRNAPHs) along a 3-km studied river. Surface water and sediment of 9 sampling campaigns were collected and analyzed using both infectivity and RT-qPCR assays. In parallel, some physico-chemical variables such as flow rate, water temperature, conductivity and total suspended solids were measured to investigate the impact of environmental conditions on phage propagation. For campaigns with low flow rate and high temperature, the results highlight a decrease of infectious phage concentration along the river, which was successfully modelled according to a first-order negative exponential decay. The monitoring of infectious FRNAPHs belonging mainly to the genogroup II was confirmed with direct phage genotyping and total phage particle quantification. Reported *k* decay coefficients according to exponential models allowed for the determination of the actual in situ distance and time necessary for removing 90 % of infectious phage particles. This present work provides a new way to assess the true in situ viral propagation along a small river. These findings can be highly useful in water quality and risk assessment studies to determine the viral contamination spread from a point contamination source to the nearest recreational areas.

## Introduction

River water is often used for the production of drinking water, irrigation or recreational areas. Therefore enteric virus occurrence in surface water generates a sanitary risk and remains a major public health issue (Ganesh and Lin 2013; Kern et al. 2013; Rodrigues et al. 2015). After their discharge in surface water, viral particles can take a variety of potential routes. Firstly, viruses spread in surface water by advection process, which refers to transport with the water flow rate; and their occurrence is affected by some environmental factors (Fong and Lipp 2005; Gantzer et al. 1998). Temperature (Bertrand et al. 2012; Lee and Sobsey 2011; Ogorzaly et al. 2010) and sunlight (Fisher et al. 2011; Kohn and Nelson 2006; Wommack et al. 1996) are among the most important stressors reducing the virus infectivity. Secondly, virus association with suspended solid particles leads to settling down to the riverbed sediment. Numerous data have already described the presence of viruses in sediment (Hargreaves et al. 2013; Leroy et al. 2008; Skraber et al. 2009) and considered this compartment as a virus reservoir (Bosch et al. 2003). In addition, a higher persistence of viruses in sediment than in the water column has been reported according to a protective effect from proteolytic enzymes or other degrading factors present in flowing water and UV inactivation (Liew and Gerba 1980; Smith et al. 1978). Finally, a mobilization of viruses accumulated in sediments can occur after natural or anthropogenic disturbances. The resuspension of sediment and subsequent release of viral particles into the water column constitute a diffuse input on contamination (Fauvel et al. 2016; Williamson et al. 2014). All of these environmental factors and processes, which govern the fate and propagation of viruses in surface water, have been relatively well documented in laboratory conditions, but very few studies have reported data on the in situ transport and spread of viruses in river (Skraber et al. 2002). In addition, another important point to consider in the monitoring of enteric viruses in surface water is the variability and diversity of interactions with surfaces and inactivation rate observed among viruses of both similar and different families. For example, several studies have already demonstrated the survival divergence of F-specific RNA bacteriophage (FRNAPH) genogroups (GGs) in water (Muniesa et al. 2009; Yang and Griffiths 2013) as well as their differing adsorption behavior with solid particles (Boudaud et al. 2012; Deboosere et al. 2012; Haramoto et al. 2015). FRNAPHs are considered good indicators to assess viral contamination due to the nature of their genome, their size, and structure that are similar to those of pathogenic viruses (Havelaar 1993). FRNAPHs belong to the *Leviviridae* family, which is divided into two genera and four GGs: *Levivirus* including GGI and GGII and *Allolevivirus* including GGIII and GGIV. FRNAPHs are ubiquitous in aquatic environments worldwide (Haramoto et al. 2009; Lucena et al. 2003; Ogorzaly et al. 2009) and therefore constitute a good tool to understand the fate of viral particles in a complex aquatic environment.

Several questions still have to be addressed to understand the fate of viral particles in a complex aquatic environment and provide useful information in the framework of water management strategies. One critical question is whether or not viruses can persist in high concentration in the water column long enough to cause disease in individuals in contact with polluted water. In other words, how long and how far from a point-source contamination can infectious viral particles travel? The *T*_90_ of FRNAPHs, time necessary to remove 90 % of infectious phages in surface water, was previously determined either via laboratory tests (Bae and Schwab 2008; Brion et al. 2002; Long and Sobsey 2004; Yang and Griffiths 2013) or in situ via dialysis sacs to permit contact of contained phages with the external aqueous milieu (Durán et al. 2002; Mocé-Llivina et al. 2005; Muniesa et al. 2009). However, no work has currently investigated the survival of viruses, directly observed in their natural media (i.e. viral particles from river, without laboratory cultivation steps) and subjected to advection process as well as environmental stressors.

The work reported here provides an original experimental approach using the residence time of the river water mass to assess the real in situ propagation of enteric viruses. The purpose of this study was to determine the in situ dynamics of the F-specific RNA bacteriophages in a small river by implementing this new method. Two main dissemination routes were investigated: (1) the phage propagation in surface water controlled by environmental variables and (2) the phage settling onto sediment.

In that context, a river stretch more than 3 km long with a unique source of fecal contamination (the effluent of a wastewater treatment plant) was selected as the ideal site for studying the fate of phage particles. The F-specific RNA bacteriophages were detected according to three distinct methods during nine experimental campaigns covering different seasons. First, the propagation of infectious FRNAPHs was modelled as a function of either time or distance along the studied river stretch. Second, genotyping of 287 plaques of infectious FRNAPHs was performed in order to characterize the in situ survival of the distinct genogroups. Third, the quantification of the total phage particles was investigated and confronted with the data of the infectious phages in order to clarify the different dissemination routes of FRNAPHs.

## Materials and Methods

### Study Area

**Fig. 1:**
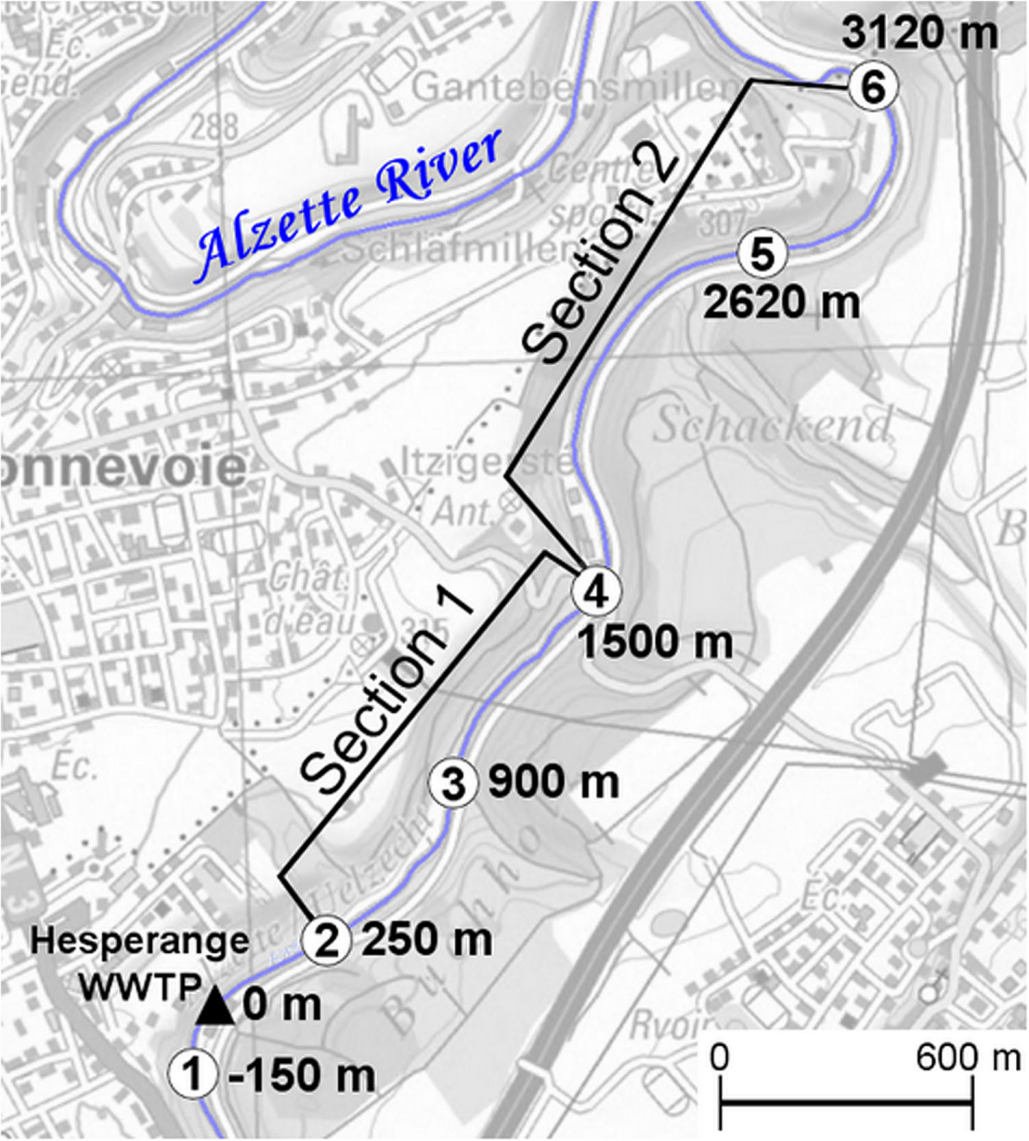
Profile of the studied river stretch. *Numbers* 1–6 indicate the six sampling sites. Conductivity monitoring point for the water residence time establishment were located at the sites 2, 4 and 6 and marked out both sections with different stream velocity

The study was conducted on the Alzette River, one of the main rivers in Luxembourg. In order to follow water mass and viral contamination, the 3.1 km-long river study site (Fig. 1) was selected based on the following conditions. On one hand, the effluent of a wastewater treatment plant (WWTP) upstream from the studied section allows for a continuous point source of fecal pollution. The presence of non-point sources, such as surface runoff or waterfowl are inherently more difficult to characterize. In order to minimize these sources, all samplings were performed under stable hydro-climatological conditions. On the other hand, the river stretch is a simple hydrographic network without dead arms. The alternation of segments with high flow velocity and slower velocity zones are supposed to ensure a good mixing of water and settling areas of suspended solids.

### Input Pollution Sources

#### Upstream River Water

Three WWTP were located upstream from the studied area with respective capacities of 24,500, 90,000 and 95,000 inhabitant-equivalents. The first WWTP was located 10 km upstream and the others were located further upstream. Fecal pollution upstream from the studied area was characterized during the nine sampling campaigns, with the collection and analysis of surface water at the site 1. An average of 1.01 ± 0.75 × 10^4^ (mean ± standard deviation) most probable number (MPN) per 100 mL of *Escherichia coli* and 8.48 ± 6.52 × 10^2^ plaque forming units (PFU) per 100 mL of infectious FRNAPHs were detected. Total phage particles were determined and revealed the presence of 7.61 ± 2.34 × 10^2^ genome copies (gc) per 100 mL for GGI, 2.74 ± 2.20 × 10^4^ gc/100 mL for GGII and 7.88 ± 3.82 × 10^2^ gc/100 mL for GGIII.

#### Hesperange Wastewater Treatment Plant

The WWTP of Hesperange constitutes the single point source of fecal pollution of the study area. This plant was renovated in 2006 and is composed of a primary treatment followed by an activated sludge plant with nitrifying moving bed biofilm reactor technology. Its maximum capacity is 26,000 inhabitant-equivalents. *Escherichia coli* and infectious FRNAPHs were detected at the outlet of the WWTP with an average (9 campaigns) of 2.59 ± 0.98 × 10^4^ MPN/100 mL and 8.86 ± 7.62 × 10^3^ PFU/100 mL, respectively. Three out of four genogroups were observed in effluents: GGI (2.68 ± 3.77 × 10^4^ gc/100 mL), GGII (2.34 ± 0.97 × 10^5^ gc/100 mL) and GGIII (4.03 ± 6.10 × 10^4^ gc/100 mL). Fecal pollution contribution of WWTP effluent in the river stretch was assessed by the comparison of *E.coli* and FRNAPH concentrations detected in surface water between upstream (site 1) and downstream (site 2) of the Hesperange WWTP. Concerning the input of *E. coli* in the river, the WWTP did not contribute significantly (Mann–Whitney rank sum test, *p* = 0.724) whereas a statistical difference was observed for infectious FRNAPHs (Mann–Whitney rank sum test, *p* = 0.042). This input of phage pollution by the WWTP effluent was particularly perceptible during campaigns performed at low flow rates (Mann–Whitney rank sum test, *p* = 0.041) compared to higher flow rates (Mann–Whitney rank sum test, *p* = 0.400) due to a dilution effect.

### Determination of Residence Time

**Fig. 2:**
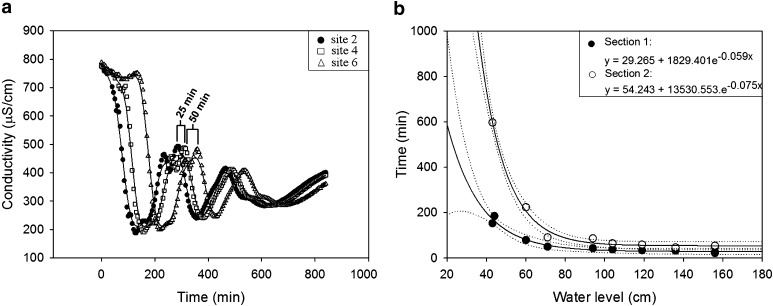
**a** Water conductivity monitoring for a high water level (156 cm at the Hesperange WWTP) for section 1 (between site 2 and 4) and section 2 (between site 4 and 6). **b** Exponential regressions (*dark lines*) of the residence time of the water body as a function of water level (95 % confidence limits in *dotted lines*). Each *point* represents an experimental measurement

In order to follow the water mass transiting along the river, the conductivity was used as a natural marker at different points of the transit sector. Three conductimeters (WTW Multi 3420) were placed along the studied stretch in accordance with the flow rate variation (Fig. 1). The whole section was therefore divided into two sections: the first one 1250 m in length with a faster flow rate (between site 2 and 4) and the second one 1620 m with a slower flow rate (between site 4 and 6). Water conductivity was monitored over several days with 15-min time gaps. Several sets of measurements were performed at different water levels to cover the variation of flow rate. Among the three conductimeter locations, similar peaks of conductivity were observed separately in time (Fig. 2a). This lag time is the time necessary of the charged water mass to cover the distance between each site. The knowledge of these times to cross sections 1 and 2 at different water levels enables the establishment of time residence equations for both sections of the Alzette River (Fig. 2b).

### Surface water and sediment sampling

In addition to upstream samples (site 1), stream water and sediment samples were collected at five sampling sites dispatched along the studied area (from site 2 to site 6) (Fig. 1). In order to have representative samples, water and sediment were taken in equal proportions from right bank, left bank, and middle of the river. The surface of the riverbed sediment was vacuumed with a pump system. Nine sampling campaigns were performed during 2014 and 2015 allowing a collection of 54 water and 42 sediment samples. Only seven out of nine campaigns (1, 2, 3, 6, 7, 8 and 9) were sampled for sediment, when the water level was lower than 60 cm. For each campaign, residence time of the water mass was determined in order to respect the travel time of water between each sampling sites (Table 3, in appendix). Samples were transported back to the laboratory in a refrigerated compartment before subsequent analysis. Sediment samples were stored for 24 h at 4 °C in order to remove excess water.

### Hydrological and Physico-Chemical Data

The flow rate (m^3^/s) of the Alzette River was estimated using the rating curve. The water level data, measured at the Hesperange WWTP, was provided by the “Administration de la Gestion de l’Eau”. Some factors, which can potentially affect the propagation of infectious FRNAPHs by either inactivating phages or influencing their interactions with environment, were evaluated for each water sample. Conductivity (µS/cm), water temperature (°C), pH and oxygen (mg O_2_/L) were measured with a portable probe (WTW Multi 3420). Total suspended solids (TSS) were quantified (mg/L) thanks to surface water filtration through glass fiber filter of 0.7 μm porosity (GF/F Whatman^®^). Finally, a measure of biochemical oxygen demand (BOD_5_), biodegradable organic matter broken down by aerobic microorganisms after 5 incubation days (mg O_2_/L), was determined (OxiTop^®^ measurement system BOD_5_).

### *Escherichia coli* Determination

The fecal indicator bacteria *E. coli* concentration was quantified in water samples using the Colilert^®^-18 Quanti-tray^®^ 2000 system (IDEXX Laboratories) and expressed in MPN/100 mL according to the manufacturer’s instructions.

### Elution of Phages From Sediment

Twenty milliliters of pyrophosphate buffer (0.01 M) at pH 7.0 were added to 10 g of wet sediment (sediment/liquid ratio = 0.5). The mixture was submitted to sonication (37 kHz) on ice for 3 min and interrupted for 30 s every minute for mixing manually the samples (Danovaro et al. 2001). Samples were then shaken at 400 rpm for 30 min at 4 °C (orbital shaker, KS 260 basic IKA^®^). Supernatant was retrieved twice after successive centrifugations at 10,000 *g* for 10 and 5 min, respectively. Ten 1-mL fractions of the supernatant were used for the determination of infectious FRNAPH concentration by plaque assay (cf. “Detection of Infectious F-Specific RNA Bacteriophages”). The recovery of the infectious FRNAPH elution step was assessed on different sediment samples by spiking a known amount of the most representative strain in sediment i.e. BZ13 strain DL20 (FJ483839) belonging to GGII. More than 10 % of infectious viral particles were found after the elution process with a recovery variability which could be influenced by the sediment matrix (29.3 ± 16.0 %, *n* = 6).

### Detection of Infectious F-Specific RNA Bacteriophages

The concentration of infectious FRNAPHs was determined using *Salmonella enteric* serovar *Typhimurium* strain WG49 and the double-agar-layer technique as described in the ISO standard 10,705-1:2001. Nalidixic acid was added to media for limiting the growth of the abundant bacterial flora. Negative and positive controls (MS2 phage) were included in all analysis. Plates were incubated overnight at 37 °C before PFU counting. FRNAPHs were enumerated in 5 × 1 mL for each water sample and their concentrations were expressed in PFU/100 mL.

### Plaque Isolation From Environmental Samples

From surface water samples collected along the 9 campaigns, 10 well-separated phage plaques formed on agar plates were randomly collected by micropipette aspiration and suspended in 1 mL of phosphate-buffered saline (PBS 0.01 M, pH 7.4, Sigma-Aldrich) supplemented with 5 % of glycerol (Ogorzaly et al. 2009). After 1 min of agitation at 30 °C (1400 rpm, Thermomixer, Eppendorf), phage isolates (287 isolates) were stored at −80 °C until characterization by molecular tools.

### Specific Detection of FRNAPH Genogroups by Real-Time RT-PCR

Genogroup-specific detection was carried out either directly on river water samples or on phages isolates obtained from the infectivity assay. In both cases, the primers/probes designed by Ogorzaly and Gantzer (2006) were selected to identify and/or quantify the four GGs, due to a spectral detection more adapted to track urban pollution (Hartard et al. 2015).

For river water samples, specific genogroup detection was performed on all water samples from the campaigns 4–9 resulting in 36 analyzed samples. A first step of clarification of surface water was performed (3000 g, 10 min) before concentration of phage particles by ultracentrifugation of 98 mL of water (Fauvel et al. 2016). After the RNA extraction (NucliSENS^®^ Magnetic Extraction kit, BioMerieux, France), genogroup quantification was performed by a two-steps RT-qPCR method (Ogorzaly and Gantzer 2006) with some modifications (Fauvel et al. 2016). Construction of standard curves with target genomic sequences of each GG was developed to determine RNA genomic concentrations. A detection limit of 10 genome copies per RT-qPCR reaction was determined for the four GGs. A negative extraction control as well as negative and positive RT-qPCR controls were incorporated into each analysis. RT-qPCR inhibition was evaluated on the 36 analyzed samples by spiking a known amount of transcribed GA plasmids into extracted samples.

For the genotyping of infectious FRNAPHs, the real-time RT-PCR was directly conducted with a One-Step RT-PCR Kit (QuantiTech Probe RT-PCR, Qiagen, France) due to the high expected concentration of phages in one plaque (Ogorzaly et al. 2009).

### Data Modelling and Statistical Analysis

The transport and propagation of FRNAPHs along the studied area was mathematically modeled. A first-order negative exponential equation was selected to fit the best to the field experiment data. The regression fit wizard was performed by the SigmaPlot 12.5 software according to the following equation: $C_{t} = C_{0} \;e^{ - kt}$, where *t* is time, *k* the first-order inactivation constant, *C*_0_ is the initial concentration of phages, and *C*_*t*_ is the concentration of phages at time *t*. In this formulation, all of the factors and processes responsible for the virus dynamics are lumped into the single *k* constant.

Other statistical analyses were carried out with XLSTAT software (Addinsoft, France). Principal component analysis (PCA) was used to get a global overview of the effect of environmental variables on the infectious FRNAPH concentration in surface water, whereas Mann–Whitney and Spearman tests were used to compare two groups of data or to relate variable correlations, respectively.

## Results

### Fate of Infectious F-Specific RNA Bacteriophages in River

#### Propagation in water column

**Fig. 3:**
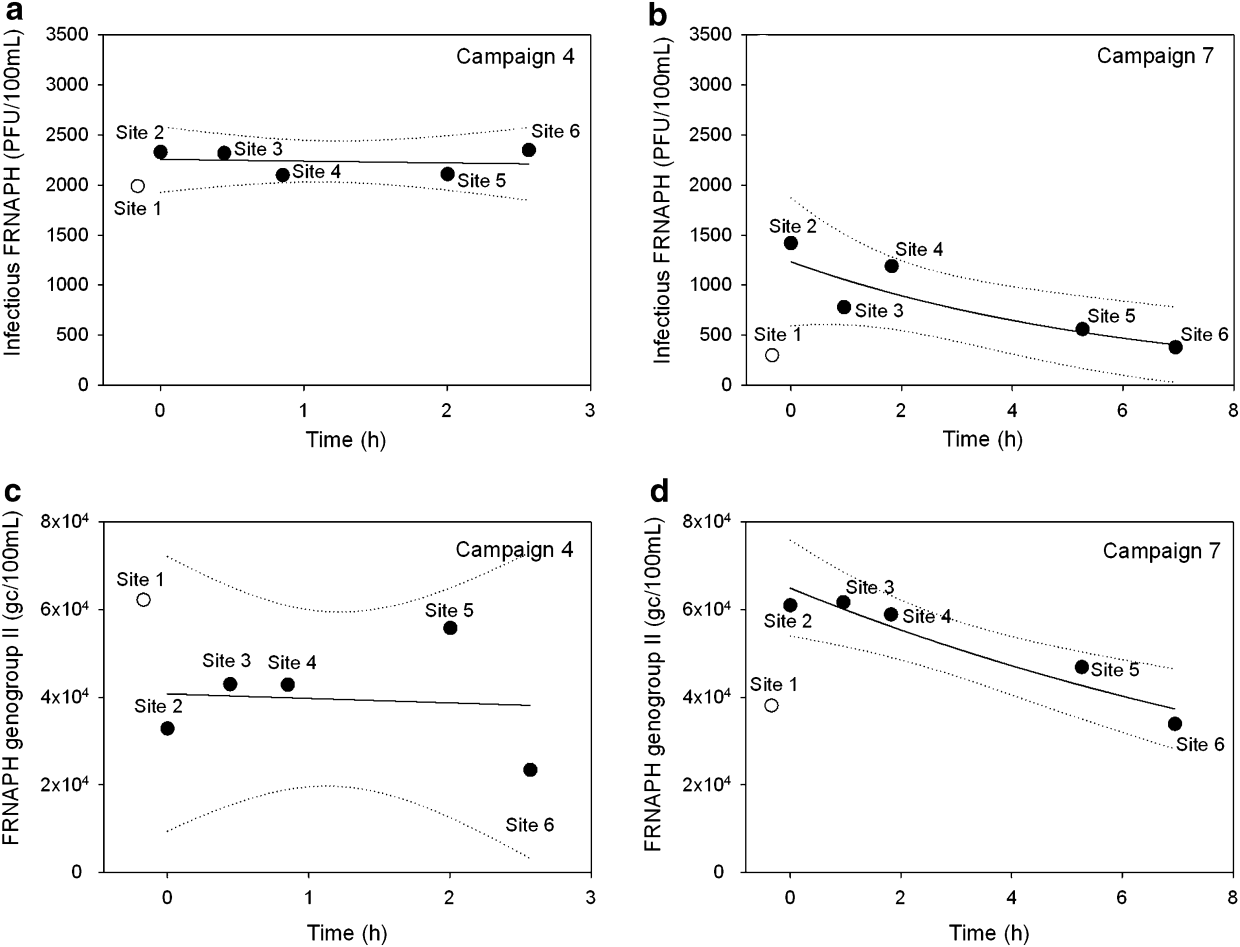
Both propagation patterns of infectious F-specific RNA bacteriophages (FRNAPH) (**a**, **b**) and total genogroup II (**c**, **d**) observed along the studied river stretch according to the residence time of the water. First-order exponential regressions are indicated by *dark lines* and 95 % confidence limits by *dotted lines*. Phage concentrations from upstream and downstream Hesperange wastewater treatment plant are represented in *white* and *black points*, respectively

Nine campaigns of field experiments were carried out between June 2014 and July 2015, each one including the monitoring of the water mass in order to follow FRNAPH propagation from a point source of fecal pollution. The time residence of the water mass for travelling over the river stretch evolved from one campaign to another according to fluctuation of the water level (Table 3, in appendix). The fate of infectious phage particles during each field campaign was modeled using FRNAPH concentration data as a first-order distance- or time-dependent decay kinetics. Two distinct patterns of FRNAPH propagation were observed (Fig. 3a, b). The first one showed a stable FRNAPH concentration all over the water course (campaigns 4, 5 and 6) whereas the other one described an exponential decay (campaigns 1, 2, 3, 7, 8 and 9). Among these decay models, one (campaign 2) was slightly different, needing the addition of a constant in the equation in order to fit to the data set ($C_{t} = a + C_{0} \;e^{ - kt}$). Indeed, infectious FRNAPH concentration from the campaign 2 presented a fast decrease between both first sites (sites 2 and 3) followed by a plateau phase along the rest of the studied stretch. These fitting exponential decay models resulted in a total of 6 validated *k* values allowing the determination of *D*_90_ (predicted distance in meter to achieve a 1−log_10_ reduction of infectious FRNAPHs) and *T*_90_ (predicted number of hour to achieve a 1−log_10_ reduction of infectious FRNAPHs) under the studied conditions (Table 1). The decay equation obtained for the campaign 2 did not allow for the calculation of *D*_90_ and *T*_90_ since 70 % of infectious FRNAPH reduction was observed between both first sites followed by a stable concentration. For the other campaigns presenting an exponential decay of phages, the estimated values of *D*_90_ and *T*_90_ varied with ranges from 2.8 to 9.5 km and from 11 h 16  to 37 h 45 min, respectively (Table 1).

**Table 1:** Data from exponential decay models describing the propagation of infectious FRNAPH along the studied area

Campaigns	Infectious F-specific RNA bacteriophages								
	Data from exponential decay models according to the distance *C*_*d*_ = *C*_0_ e^−*kd*^				Data from exponential decay models according to the time *C*_*t*_ = *C*_0_ e^−*kt*^				Log_10_ reduction^b^
	*C*_0_	*k*	*R* ^2^	*D*_90_ (m)	*C*_0_	*k*	*R* ^2^	*T*_90_ (h:min)	
1	1447.69	8.20E−04	0.996	2808.0	249.68	0.204	0.907	11:16	−0.99
2^a^	1939.2	3E−3	0.968	×	1939.0	1.275	0.968	x	−0.41
3	1330.94	3.80E−04	0.887	6059.4	1156.38	0.096	0.970	23:57	−0.45
4	×	0	×	×	×	0	×	×	0.00
5	×	0	×	×	×	0	×	×	0.03
6	×	0	×	×	×	0	×	×	−0.03
7	1447.69	3.85E−004	0.676	5985.4	1233.12	0.161	0.716	14:18	−0.57
8	3470.98	2.43E−004	0.984	9479.6	3075.21	0.061	0.967	37:45	−0.31
9	1709.47	3.25E−004	0.654	7091.4	1547.00	0.082	0.841	28:05	−0.39

^a^Equation of campaign 2 exponential decay model was different ($C_{t} = a + C_{0} \;{\text{e}}^{ - kt}$) and did not allow for the calculation of *D*_90_ or *T*_90_

^b^Log_10_ reduction of infectious F-specific RNA bacteriophage concentrations between site 2 and site 6

#### Occurrence in Sediment

All of the 42 fresh sediment samples were positive for the presence of infectious FRNAPHs. No propagation pattern was observed along the studied area with a random variation of phage concentrations from one site to another (Fig. 6, in appendix). Indeed, an important dispersion occurred among all samples with an average of 96.8 ± 125.1 PFU/g of dry sediment and a minimum and maximum concentration of 0.7 PFU/g and 495.6 PFU/g, respectively.

### Fate of Total Phage Particles in Surface Water

In addition to the monitoring of the infectious FRNAPHs, analysis of each FRNAPH GG was independently conducted with the RT-qPCR system developed by Ogorzaly and Gantzer (2006). All samples were inhibited at less than 20 % excepted for samples of the campaign 4 with 58 ± 6 % of inhibition. Among the four GGs, GGII was noted to be the most abundant in the 36 river water samples with 100 % of detection and a range from 4.7 × 10^3^ to 6.3 × 10^4^ gc/100 mL. Other GGs were not systematically detected with 33.3, 44.4 and 88.8 % of samples under the detection limit for GGI, GGIII and GGIV, respectively. The GGI (from 9.3 × 10^2^ to 6.1 × 10^4^ gc/100 mL) was observed more often than GGIII (from 3.2 × 10^2^ to 1.7 × 10^4^ gc/100 mL) and GGIV (from 3.1 × 10^2^ to 6.2 × 10^2^ gc/100 mL). Concentration variation among water samples can be due to the variation of GG discharges at the outlet of the Hesperange WWTP. The propagation modelling for GGI, GGIII and GGIV was precluded due to their low level of detection. However, for GGII, two distinct patterns of propagation were observed (Fig. 3c, d). For the campaigns 4, 5, 6 and 8, a weak random variation of GGII concentrations from one site to another occurred inducing a failure of the decay modelling, whereas an exponential decrease was observed along the studied area for both others campaigns (7 and 9). Both *k* values obtained (*k* = 0.080, *R* ^2^ = 0.886 and *k* = 0.015, *R* ^2^ = 0.626 for campaigns 7 and 9, respectively) were lower than those calculated for the infectious FRNAPHs. From these results, the GGII total phage particles seem to be less affected by environmental stressors or settling process than infectious FRNAPHs.

### Effect of Environmental Factors on Infectious FRNAPHs

The effects of flow rate, conductivity, pH, water temperature, oxygen, TSS and BOD_5_ on the infectious FRNAPH concentrations in river water were investigated. Water temperature (directly measured) and microbial activity (assessed with measurements of oxygen and BOD_5_) can influence the survival of infectious FRNAPHs; whereas chemical composition (assessed with oxygen, pH and conductivity measurements) or presence of organic matter (assessed with BOD_5_) can take part into the attachment of phage particles to TSS.

**Fig. 4:**
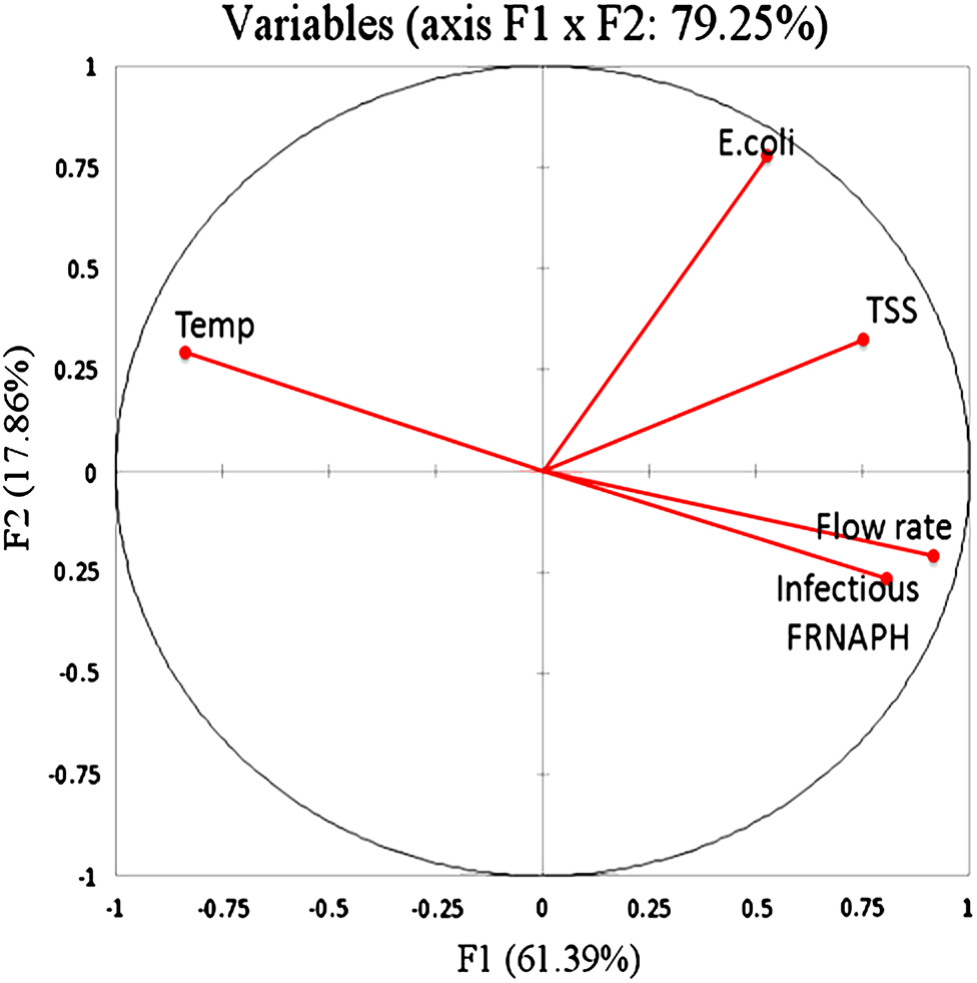
Principal component analysis of the physico-chemical and microbiological parameters measured on water samples collected from site 2 to 6 during the 9 campaigns (*n* = 45). *TSS* total suspended solids; *Temp* water temperature; *FRNAPH* F-specific RNA bacteriophages

Conductivity, pH, oxygen and BOD_5_ were stable over the 9 campaigns. The chemical composition of surface water and the presence of organic matter do not seem associated with the variability of microbiological measurements (infectious FRNAPHs and *E. coli*). On the contrary, flow rate, water temperature and TSS varied (Fig. 7, in appendix). The relationships between these latter parameters, *E. coli* and infectious FRNAPHs were studied by PCA (Fig. 4). More than 79.25 % of the total information in the data can be explained with this PCA (PC1 61.39 %, PC2 17.86 %) and all of the variables considered are well represented with respect to the correlation circle. Table 2 presents the correlation matrix between all variable parameters. First, inability of fecal indicator bacteria to assess viral contamination in aquatic environment was shown by the absence of correlation between *E. coli* and infectious FRNAPHs (Spearman correlation test, *r* = 0.286, *p* = 0.057). Then, correlations were observed between infectious phages and other variables.

**Table 2:** Correlation matrix (Spearman correlation coefficient and *p* values)

Variables	Temperature	TSS	*E. coli*	Infectious FRNAPHs	Flow rate
Temperature	1				
TSS	−0.433 *p* = 0.003	1			
*E. coli*	−0.321 *p* = 0.032	0.444 *p* = 0.002	1		
Infectious FRNAPHs	−0.678 *p* < 0.0001	0.394 *p* = 0.008	0.286 *p* = 0.057	1	
Flow rate	−0.822 *p* < 0.0001	0.602 *p* < 0.0001	0.346 *p* = 0.020	0.698 *p* < 0.0001	1

*TSS* total suspended solids; *FRNAPHs* F-specific RNA bacteriophages

On one hand, total suspended solid was weakly positively correlated with infectious FRNAPHs (*r* = 0.394, *p* = 0.008). If phage particles are attached to TSS, they should present the same dynamic along the river stretch. A comparison of TSS and infectious FRNAPHs between the beginning (site 2) and the end (site 6) of the studied river section highlighted a different dynamic of each suspended particles. Whatever the external conditions, TSS concentrations along the studied river remained stable with a low range of log_10_ reduction (from 0.12 to −0.22) between sites 2 and 6. Whereas propagation of infectious FRNAPHs varied depending on the external conditions with a high range of log_10_ reduction (from 0.03 to −0.99).

On the other hand, infectious phages were more significantly positively correlated with flow rate (*r* = 0.698, *p* < 0.0001) and negatively with water temperature (*r* = −0.678, *p* < 0.0001) (Table 1). In addition, important variations of flow rate and water temperature were measured during the 9 campaigns (from 0.53 to 4.16 m^3^/s and from 3.7 to 20.3 °C, respectively). According to these results and both different infectious FRNAPH patterns (cf. “Propagation in Water Column”), “high flow–low temperature” (campaigns 4, 5 and 6 where flow rate was higher than 1.99 m^3^/s and water temperature lower than 8 °C) and “low flow–high temperature” (campaigns 1, 2, 3, 7, 8 and 9 where flow rate was lower than 1.45 m^3^/s and water temperature higher than 11 °C) conditions seem to have an effect on phage dynamic in the river.

### Impact of Environmental Conditions on Infectious F-Specific RNA Bacteriophage Genogroup Repartition

**Fig. 5:**
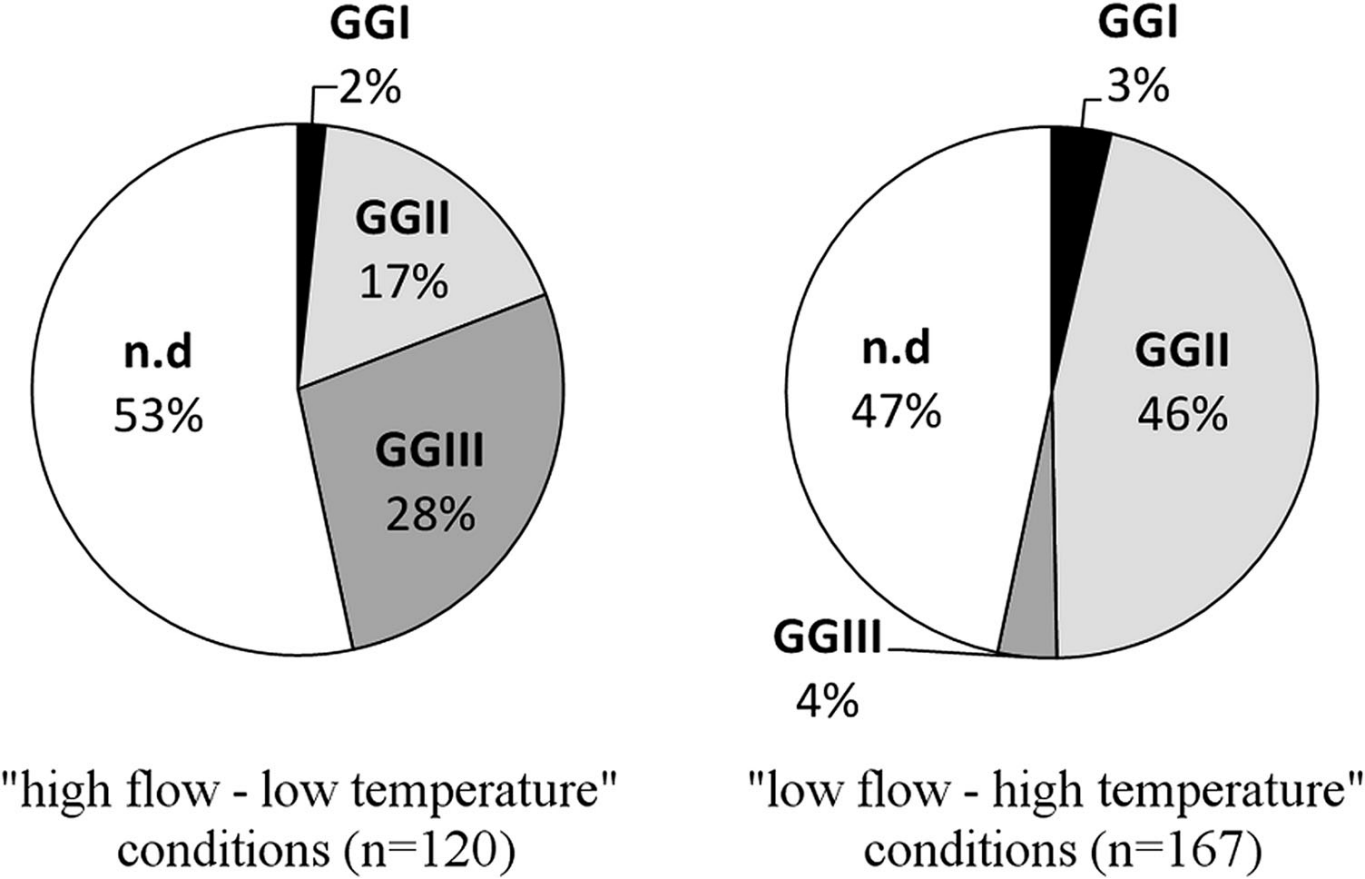
Distribution of the four genogroups (GG) of infectious F-specific RNA bacteriophages in surface water according to environmental conditions. *nd* Not detected with selected primers (Ogorzaly and Gantzer 2006)

**Fig. 6:**
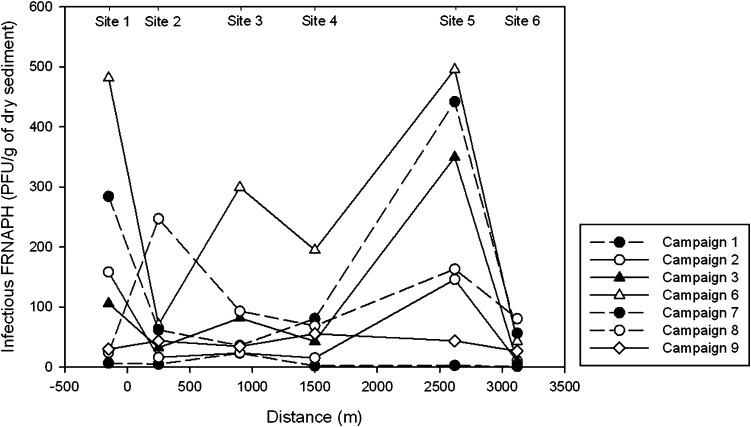
Concentration of infectious F-specific RNA bacteriophages (FRNAPH) in sediment samples at the 6 sampling sites during 7 campaigns

**Fig. 7:**
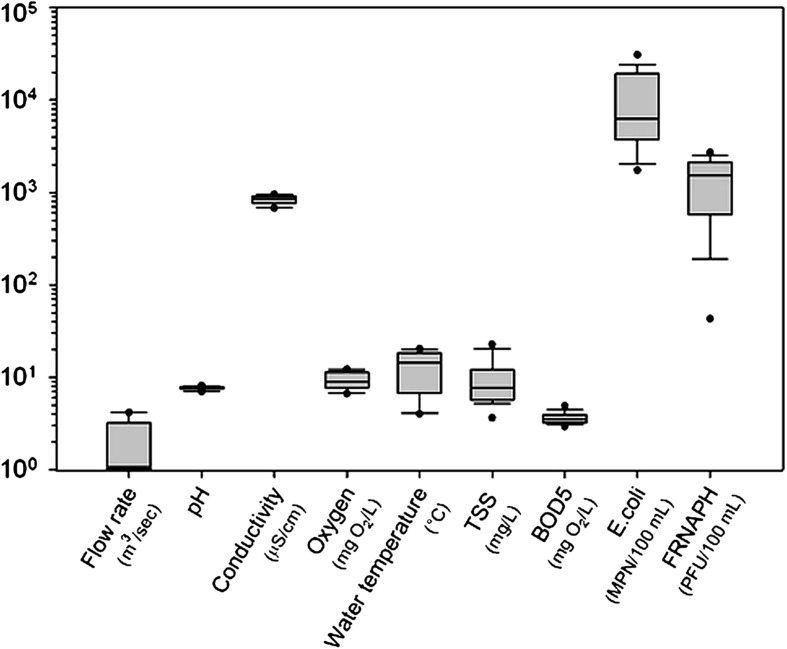
Dispersion of the physico-chemical and microbiological parameters measured during the 9 sampling campaigns from site 2 to site 6 (*n* = 45). *Box* 25 and 75th percentiles, *whisker* 10 and 90th percentiles, *horizontal line* within the *box* median, *dot* minimum and maximum values. *TSS* total suspended solids; *BOD*_*5*_ biochemical oxygen demand after 5 incubation days; *FRNAPH* F-specific RNA bacteriophages

Genogroup-specific detection carried out on 287 isolates showed different GG distributions according to environmental conditions (Fig. 5). Out of the 120 plaques isolated from “high flow–low temperature” conditions, 28 % were identified as belonging to GGII, 17 % to GGIII and 2 % to GGI. The distribution was different for “low flow–high temperature” conditions with a more important proportion of GGII (46 %) and less GGIII detected (4 %). In both cases, the proportion of not detected samples (53 and 47 % for both respective conditions) can be assimilated to a non-urban pollution depending on the choice of primers/probes (Hartard et al. 2015). Nevertheless, the affiliation to the *Leviviridae* family of 108 out of 142 non detected isolates was examined with a RNase test (ISO 10705-1). The addition of the RNase enzyme allows distinguishing FRNAPHs and F-specific DNA bacteriophages (FDNAPHs) (*Inoviridae* family). Each isolate suspension spotted onto plates containing *Salmonella typhimurium* strain WG49, which were not able to develop in presence of RNase activity, was confirmed to be FRNAPHs. Among the isolates tested with RNase, 70 % of them were confirmed to be FRNAPHs, 22 % were FDNAPHs and 8 % of plaques were not developed with or without nucleases.

## Discussion

The objective of this study was to determine the in situ dynamics of F-specific RNA bacteriophages in a small river. To this purpose, an original experimental approach considering the residence time of the river water mass was applied along five sampling sites on the Alzette River. F-specific RNA bacteriophages were screened in surface water and sediment along nine sampling campaigns. Two principal dissemination routes were considered: (1) the spread of FRNAPHs in surface water controlled by environmental variables and (2) the settling onto sediment.

The transport of suspended particles related to water flow was the first way to consider in the phages’ propagation. Infectious FRNAPHs were detected in the Alzette River with concentrations ranging from 1.5 to 3.5 log_10_ PFU/100 mL, which was comparable to the results of previous studies (Lucena et al. 2003; Ogorzaly et al. 2009; Skraber et al. 2009). The analysis of all data collected from the nine campaigns emphasized that the variability of infectious FRNAPH concentrations was significantly correlated to flow rate and water temperature. For campaigns with high flow rates and low temperatures, the concentration of infectious FRNAPHs globally remained stable, whereas for campaigns with lower flow rates and higher temperatures, phage concentrations were lower and decreased along the river. The increase of flow rate is often associated with an increase of phages in surface water, due to the capability of the higher velocity to resuspend riverbed sediments (Fauvel et al. 2016), whereas an increase of water temperature is often associated with inactivation of infectious phages in surface water (Bae and Schwab 2008; Yang and Griffiths 2013). Therefore, in addition to the propagation of infectious FRNAPHs in surface water by advection processes, results reveal a natural inactivation of bacteriophages related to water temperature. The impact of the seasonal temperature (summer vs winter) on the survival of infectious FRNAPHs was previously shown (Durán et al. 2002; Hata et al. 2016). In order to characterize the in situ survival of the distinct genogroups of infectious FRNAPHs, genotyping of 287 individual infectious phage plaques was carried out. The distribution of GGII and GGIII differed according to flow rate and water temperature conditions. Infectious FRNAPHs belonging to GGIII were detected in lower proportion during “low flow–high temperature” than during “high flow–low temperature” in favor of GGII. The greater survival at high temperature of GGII compared to GGIII was previously reported (Long and Sobsey 2004). This view confirms previous observations regarding the differential persistence of FRNAPH GG in surface water (Muniesa et al. 2009; Schaper et al. 2002). Furthermore, the occurrence of infectious FRNAPHs belonging especially to GGII and GGIII highlight the anthropogenic pressure on the studied area. Indeed, FRNAPHs are often proposed as tracers to distinguish human (GGII and GGIII) from non-human (GGI and GGIV) fecal contamination sources (Lee et al. 2009, 2011). This predominance of an urban pollution in the Alzette River stretch is also confirmed by the abundance of total phage particles GGII directly quantified in water samples by RT-qPCR. Thus, the in situ propagation of infectious FRNAPHs in the studied river stretch mainly describes the behavior of infectious GGII phages.

As far as campaigns with low flow rate and high temperature are concerned, the transport of infectious FRNAPHs was successfully modeled according to a first-order exponential decay. This approach was already chosen by other authors as the best way to explain the dynamic of a microbial population in a small river (Beaudeau et al. 2001). Reported decay coefficients, *k* values, varied from 0.082 to 0.204 with the highest value of 1.275 for campaign 2. This single *k*-coefficient lumped together environmental stressors and processes responsible for infectious FRNAPH dynamics (Jamieson et al. 2004). The precedent described results highlighted the effect of flow rate and water temperature on the variability of infectious FRNAPHs. However, the *k* values obtained from models were not significantly correlated to temperature solely (Mann–Whitney rank sum test, *p* = 0.059) or flow rate solely (Mann–Whitney rank sum test, *p* = 0.005). Considering the *k* values, the monitoring of in situ phage propagation underlined that despite the predominance of environmental stressors such as water temperature and flow rate, the combination of all environmental factors explain the phage dynamics. The intricacy of the environmental factor association was previously demonstrated. For example, Kutz and Gerba (1988) showed the different inactivating effect of the same temperature conditions on an enteric virus according to the type of water (tap water, polluted or unpolluted river water, etc.). This outcome underlined the combined impact of temperature and water chemical components on virus survival. The experimental approach developed in the present study therefore constitutes an interesting tool to assess the in situ survival of FRNAPHs in a river by taking into account the complexity of numerous environmental factors.

From exponential decay models of infectious FRNAPHs, corresponding *T*_90_ values were estimated to be ranging between 11 and 38 h in the studied river stretch. These values of *T*_90_, estimated for a majority of infectious phages belonging to GGII, are globally lower than in other studies. Indeed, for strains belonging to GGII, Brion et al. (2002) and Yang and Griffiths (2013) have determined, in laboratory experiments, *T*_90_ values of 1.5 days to 4 days at 25 °C, respectively. Muniesa et al. (2009) have meanwhile shown through the use of dialysis bags a *T*_90_ of about 3 days for GGII phages in river water at 20 °C. The different characterization of the survival time of infectious FRNAPHs in these previous studies, either through laboratory tests or through the use of dialysis bags, compared to this work, can explained shorter *T*_90_ estimates of infectious FRNAPHs. This present work provides the first investigation on the real in situ survival of viruses, directly originating from the river and subjected to the combination of flow rate and environmental stressors.

In order to further enhance the determination of the in situ dynamics of FRNAPHs in the river, the settling of infectious FRNAPHs to sediment was the other main investigated dissemination route. The presence of infectious FRNAPHs in the sediment compartment confirms the settling of phage particles from surface water towards the sediment. However, no propagation pattern was observed in sediment samples along the studied area, phage concentrations varying randomly from one site to another. In the surface water compartment, a decrease of phage concentration from the pollution point source (WWTP) took place under “low flow–high temperature” conditions. In addition to infectious phage inactivation, the settling to sediment could also explain this reduction. In order to test this hypothesis, the monitoring of total phage particles was carried out in the surface water compartment. If only inactivation is responsible for the reduction of infectious FRNAPHs along the river, total phage particles (estimated by viral RNA quantification) have to remain stable in the water column. Whereas, if phage reduction is mainly due to the settling to sediment, a reduction of total phage particles would also be observed. In this study, only the propagation of total phage particles belonging to GGII could be determined along the river stretch. The modelling for GGI, GGIII and GGIV was precluded due to their low level of detection. No significant decrease of FRNAPH GGII was observed under “low flow–high temperature” conditions with a weak random variation from one sampling site to another. Exceptions occurred for campaigns 7 and 9 with a weak decrease of total GGII phages. Nevertheless, this slight reduction of the total GGII phage particles compared to infectious FRNPAHs for these same campaigns did not permit confirming the settlement of phage particles. These results confirm on one hand the longer persistence of viral genomes compared to the infectious status of the corresponding virus (Bertrand et al. 2012; Gassilloud et al. 2003) and on the other hand the low impact of sedimentation. Finally, the stability of TSS concentrations disproves also the hypothesis of sedimentation as being significantly responsible for the decrease of FRNAPHs throughout the course of the river stretch. These last results highlighted the slow rate of the suspended particles sedimentation process, potentially undetectable in the time scale covered by the water mass transit along the studied sites.

To conclude, the innovative use of the residence time of the water mass enabled an accurate study of the in situ dynamics of FRNAPHs in a small river. First, the detection of FRNAPHs in three different ways allowed characterizing the main dissemination routes of the viral particles. The inactivation of infectious FRNAPHs was the major process of the in situ viral particle decrease along the river stretch whereas the settling onto sediment does not seem significantly responsible. It will be interesting to study the in situ dynamic of FRNAPHs in a longer section than the one selected here in order to overview both the propagation of viral particles in surface water and their transfer to sediment. Secondly, in addition to the determination of the real in situ survival rate of infectious phages (*T*_90_), this original phage monitoring described here allowed the estimation of the distance necessary to remove 90 % of infectious FRNAPHs (*D*_90_). In the studied river stretch, *D*_90_ values were estimated to be ranging from 2.8 km up to 9.5 km. In the literature, no data is currently available to compare these *D*_90_ values since this is the first time that the in situ transport of a viral pollution is investigated. Since FRNAPHs can be considered as adequate model organisms for enteric viruses (Havelaar et al. 1993), these *D*_90_ values could be interesting data to complete risk assessment models. This new method of viral propagation monitoring can therefore be very useful in water quality and risk assessment studies for determining the spread of viral pollution from a point contamination source to the nearest recreational areas.

## Appendix

See Figs. 6 and 7 and Table 3.

**Table 3:** Time residence of the water mass and stream velocity for sections 1 and 2 of the studied river during the 9 campaigns

Number of sampling campaign	Date	Water level (cm)	Time residence section 1 (min)	Stream velocity section 1 (m/s)	Time residence section 2 (min)	Stream velocity section 2 (m/s)
1	06/24/2014	42	183	0.11	634	0.04
2	01/07/2014	42	183	0.11	634	0.04
3	07/16/2014	44	166	0.12	553	0.05
4	01/14/2015	75	51	0.41	103	0.26
5	02/12/2015	71	57	0.36	120	0.22
6	03/17/2105	58	89	0.23	229	0.12
7	04/21/2105	53	109	0.19	308	0.09
8	06/03/2015	45	158	0.13	517	0.06
9	07/14/2015	43	174	0.12	592	0.05
